# Growth of catalyst-free high-quality ZnO nanowires by thermal evaporation under air ambient

**DOI:** 10.1186/1556-276X-7-220

**Published:** 2012-04-13

**Authors:** Ping Liu, Yanbin Li, Yanqing Guo, Zhenhua Zhang

**Affiliations:** 1 School of Electric and Information Engineering, Zhongyuan University of Technology, Zhengzhou 450007, China

**Keywords:** zinc oxide, nanowire, thermal evaporation

## Abstract

ZnO nanowires have been successfully fabricated on Si substrate by simple thermal evaporation of Zn powder under air ambient without any catalyst. Morphology and structure analyses indicated that ZnO nanowires had high purity and perfect crystallinity. The diameter of ZnO nanowires was 40 to 100 nm, and the length was about several tens of micrometers. The prepared ZnO nanowires exhibited a hexagonal wurtzite crystal structure. The growth of the ZnO nanostructure was explained by the vapor-solid mechanism. The simplicity, low cost and fewer necessary apparatuses of the process would suit the high-throughput fabrication of ZnO nanowires. The ZnO nanowires fabricated on Si substrate are compatible with state-of-the-art semiconductor industry. They are expected to have potential applications in functional nanodevices.

## Background

In the past decade, significant interest has emerged in the synthesis of one-dimensional semiconductor materials, such as Si [1-3], SiC [4,5], GaN [6-8], SnO_2 _[9] and ZnO [10-13]. Among these nanoscale semiconductors, ZnO has attracted a great deal of attention because of its potential as a large direct band gap semiconductor (Eg is about 3.35 eV at room temperature) with high exciton binding energy (60 meV). It can act as building blocks for nano-FET, nanolasers, photodetectors and gas sensors [8,14]. In addition, ZnO nanowires have excellent field emission for its good hardness, thermal stability and resistance to oxidation [15,16].

Recently, many methods have been developed to synthesize ZnO nanowires, for example, carbon thermal reduction [13,17], chemical vapor deposition [12,18], physical vapor deposition [19], electrodeposition [20], aqueous synthesis [21] and solvothermal technique [22]. In this paper, we synthesized ZnO nanowires by thermal evaporation without a catalyst under air ambient. The reactions were carried out in a traditional horizontal furnace with one end open at 750°C. The gray-white product was successfully deposited on the Si substrate. The process does not need any metal catalyst, so it avoids catalyst contamination. Furthermore, the simplicity, low cost and fewer necessary apparatuses of the process would suit the high-throughput fabrication of ZnO nanowires.

## Methods

**Figure 1:**
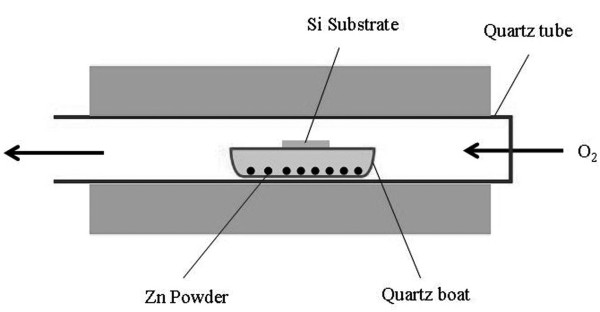
**Schematic diagram of the experimental setup for synthesizing ZnO nanowires**.

The experiments were conducted in a horizontal furnace as schematically outlined in Figure 1. The raw Zn material (99.99%) was loaded into a quartz boat. The Si substrate was cleaned by the standard cleaning process, and then, it was laid above the Zn powders. The furnace was heated to 750°C under a constant flow of pure O_2 _gas, with the flow rate of 2 ml/min. Afterwards, the quartz boat was put in the central region of the horizontal quartz tube. After 2 h, the furnace was turned off and naturally cooled to room temperature. A gray-white layer was coated on the Si Substrate.

The as-synthesized products were characterized by X-ray diffraction (XRD) with CuKα radiation (wavelength, λ = 1.5406 Å), field emission scanning electron microscopy (SEM) (Hitachi S-4800, Hitachi Ltd., Tokyo, Japan), transmission electron microscopy (TEM) and high-resolution transmission electron microscopy (HRTEM) (JEOL JEM2010F, JEOL Co., Ltd., Beijing, China).

## Results and discussion

**Figure 2:**
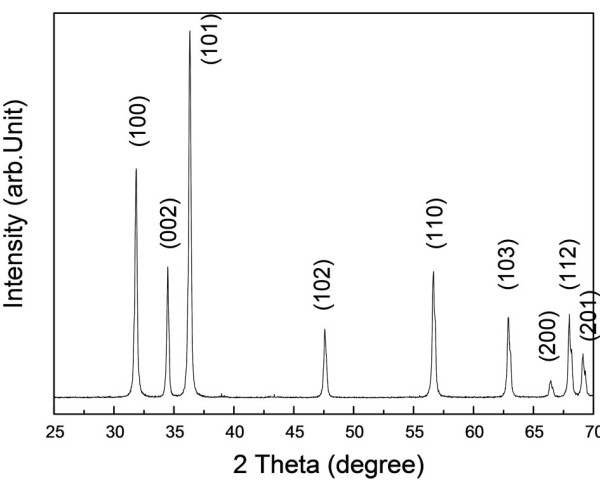
**XRD pattern of the as-synthesized products**.

Phase analysis of as-synthesized products is shown in Figure 2. All the diffraction peaks can be indexed as wurtzite ZnO with lattice constants of *a *= 0.325 nm and *c *= 0.521 nm, agreeing well with the calculated diffraction pattern (JCPDs card no.03-1005). No other diffraction peaks are detected in the spectrum within the instrumental resolution, which indicates that the products are ZnO with high purity.

**Figure 3:**
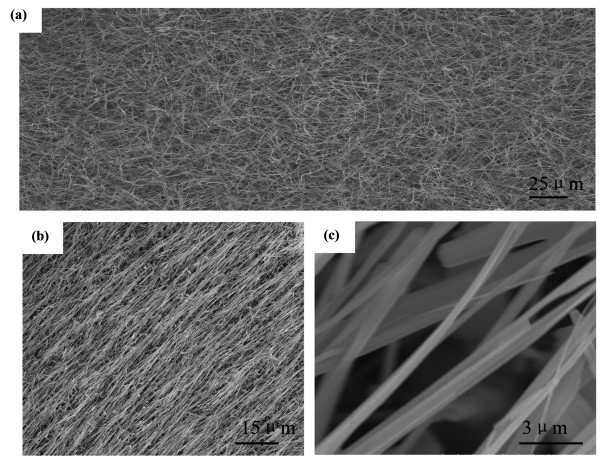
**SEM images of as-synthesized ZnO nanowires**. **(a) **Low-magnification, **(b) **aligned ZnO nanowires and **(c) **its larger-magnification Figure 4a shows the typical low-magnification TEM image of the prepared ZnO nanowire, which indicates that the ZnO nanowire has a uniform diameter of 45 nm. The as-prepared ZnO nanowire was further analyzed with HRTEM, as shown in Figure 4b. The measured spacing of lattice fringes is 0.52 nm, corresponding to the d-spacing of the (0001) planes of wurtzite ZnO. Based on the HRTEM image of the nanowire, no stacking faults and dislocations are observed. This reveals the well-crystalline nature of ZnO nanowire. In this work, highly crystalline ZnO nanowires are synthesized in the absence of a catalyst.

Figure 3a shows the typical low-magnification SEM images of the products on the Si substrate. It reveals that the products consist of high-density ZnO nanowires with typical lengths in the range of several tens of micrometers. Some aligned ZnO nanowires were also detected, as shown in Figure 3b. Figure 3c shows the larger magnification of the aligned ZnO nanowires. They reveal that the ZnOs with a diameter of 40 to 100 nm were nearly parallel and have a smooth surface. In order to investigate the further structure of the fabricated ZnO nanowires, TEM and HRTEM analyses were also performed.

**Figure 4:**
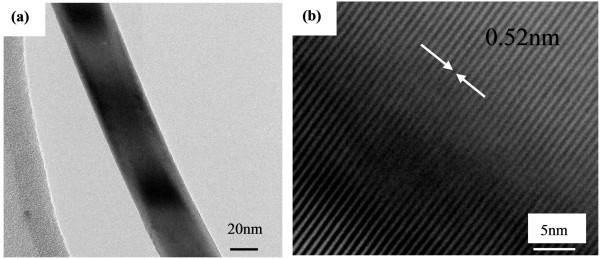
**TEM (a) and HRTEM (b) images of as-synthesized ZnO nanowire**.

The vapor-liquid-solid (V-L-S) and vapor-solid (V-S) formation mechanisms are usually responsible for the one-dimensional (1-D) semiconductor nanowires. For the V-L-S mechanism, the metal nanoclusters act as catalyst and guide the nanowire to grow towards the 1-D direction. It is evident that the nanowire tip will have an alloy droplet. In our experiment, no impurity metal particles were detected in the ZnO nanowires (from the SEM and TEM images). Hence, the V-S growth process could be well accepted in our work. Firstly, a reaction occurs between Zn and O_2 _to form ZnO_x _(x < 1); the ZnO_x _vapor is transferred by the O_2 _to the nuclei at the Si substrate. The continuously introduced O_2 _oxidizes ZnO_x _to ZnO. Due to the high supersaturation of Zn, ZnO_x _vapor and oxygen, the ZnO nanostructures can easily nucleate and grow along the [0001] direction, which was substantiated by the HRTEM image (Figure 4b). The already-formed ZnO nucleation continues to grow along the direction of the O_2 _gas flow, so some aligned ZnO nanowires on the Si substrate are formed.

## Conclusions

ZnO nanowires with high purity and perfect crystallinity were fabricated by simple thermal evaporation of pure Zn powders under air ambient without any catalyst. The diameter of the ZnO nanowires was 40 to 100 nm, and the length was about several tens of micrometers. Some aligned ZnO nanowires with smooth surface were also detected. The growth of ZnO nanostructure was explained by the V-S mechanism. The prepared ZnO nanowires exhibited a hexagonal wurtzite crystal structure. The as-fabricated ZnO nanowires are expected to find applications in nanosensors and nanodetectors.

## Competing interests

The authors declare that they have no competing interests.

## Authors' contributions

PL prepared the manuscript and supervised all of the study. YBL performed the experiment. YQG helped in the technical support for the experiments. ZHZ participated in the measurements. All the authors discussed the results and approved the final manuscript.

## Authors' information

Dr. Ping Liu got her PhD degree in 2010. She has devoted her effort in the research of one-dimensional semiconductor materials for 7 years. Her research interest lies in the fabrication and application of zinc oxide nanowires. She has published her work in several important international journals.
